# A Histologically Diagnosed Case with Infantile Osteopetrosis Complicated by Hypopituitarism

**DOI:** 10.1155/2015/786836

**Published:** 2015-10-20

**Authors:** Gulden Diniz, Ozgur Olukman, Sebnem Calkavur, Muammer Buyukinan, Canan Altay

**Affiliations:** ^1^Department of Pathology, Dr. Behcet Uz Children's Hospital, Alsancak, 35220 Izmir, Turkey; ^2^Department of Neonatology, Dr. Behcet Uz Children's Hospital, Alsancak, 35220 Izmir, Turkey; ^3^Department of Pediatric Endocrinology and Metabolism, Dr. Behcet Uz Children's Hospital, Alsancak, 35220 Izmir, Turkey; ^4^Department of Radiology, Dr. Behcet Uz Children's Hospital, Alsancak, 35220 Izmir, Turkey

## Abstract

Malignant infantile osteopetrosis is a rarely seen severe disorder which appears early in life with general sclerosis of the skeleton. It is caused by functionally defective osteoclasts which fail to resorb bone. Affected infants can exhibit a wide spectrum of clinical manifestations including impaired hematopoiesis, hepatosplenomegaly, visual impairment, and hypocalcemia. With the exception of secondary hyperparathyroidism, involvement of the endocrine system seems to be quite rare. Hypopituitarism is defined as underproduction of the growth hormone in combination with deficiencies of other pituitary hormones. Any lesion that damages hypothalamus, pituitary stalk, or anterior pituitary can cause secondary hypopituitarism. In this report, we presented a rare combination of malignant infantile osteopetrosis and secondary hypopituitarism in a newborn who presented predominantly with endocrinological symptoms. This is the first case report of malignant infantile osteopetrosis accompanied by hypopituitarism secondary to sclerosis of the sella turcica. On the other hand, this is a very interesting case which was diagnosed based on histological examination of bone marrow biopsy specimens despite lack of any clinical suspicion.

## 1. Introduction

Malignant infantile osteopetrosis (MIOP) is a rarely encountered recessively inherited autosomal disorder which presents early in life with general sclerosis of the skeleton [1]. Its incidence is 5/1,000,000 live births without any gender predisposition [1, 2]. The disease presents in the first few months of life with the manifestations probably relating to underlying defect of osteoclastic bone resorption and the most debilitating consequences develop in the skeletal, hematopoietic, and nervous systems. Affected infants can exhibit a wide spectrum of clinical symptoms including impaired hematopoiesis, hepatosplenomegaly, visual impairment, and hypocalcemia. With the exception of secondary hyperparathyroidism, involvement of the endocrine system seems to be quite rare [3, 4].

Hypopituitarism is defined as underproduction of the growth hormone alone or in combination with deficiencies of other pituitary hormones. Thanks to expanding knowledge about the genes that direct pituitary development or hormone production, an increasing proportion of cases can be attributed to specific genetic disorders. On the other hand, any lesion that damages the hypothalamus, pituitary stalk, or anterior pituitary can cause secondary hypopituitarism [5].

To our knowledge, this is the first report of MIOP accompanied by neonatal hypopituitarism secondary to sclerotic dysplasia of the sella turcica.

## 2. Case Report

A female term newborn baby delivered at 38th gestational week was admitted to our hospital because of respiratory distress and dysmorphic features. There was no history of parental consanguinity or any other inherited genetic disorder in her family. On initial presentation, she was characterized by macrocephaly, bulging anterior fontanel, small nose, flat nasal bridge, downward slanted palpebral fissures, low-set malformed ears, high arched palate, and hypotonia. Tachypnea, tachycardia, and central cyanosis were remarkable. The baby was intubated and started on conventional mechanical ventilation after detecting respiratory acidosis based on arterial blood gas analysis. On her postnatal 48 hours she developed tonic seizures which responded to phenobarbital treatment. On follow-up, arterial blood gas levels were normalized, respiratory distress symptoms were significantly ameliorated, and pulmonary hypertension was relieved without any evidence of further seizure activity. Then the infant was weaned from mechanical ventilation within 7 days. On day 17 she experienced a second episode of tonic seizure accompanied by apnea and bradycardia. Subsequent physical examination revealed hepatosplenomegaly. Routine laboratory findings demonstrated anemia, thrombocytopenia, hypoglycemia, prolonged unconjugated hyperbilirubinemia, and elevated serum lactate dehydrogenase levels (Table 1). Results of metabolic screening tests were completely normal and viral serological markers were negative.

**Figure 1:**
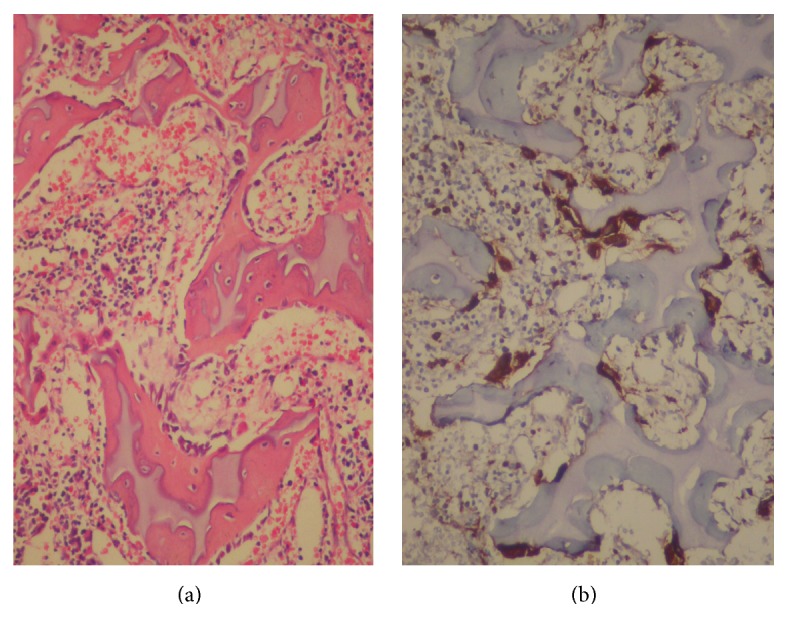
(a) Bone marrow biopsy: hypocellular bone marrow, enlarged and thickened bone trabeculae with encroachment on marrow spaces, and a mosaic pattern with cartilaginous islands (HE ×100) and (b) TRAP staining of bone biopsy specimens: increased number of osteoclasts (DAB ×200).

**Figure 2:**
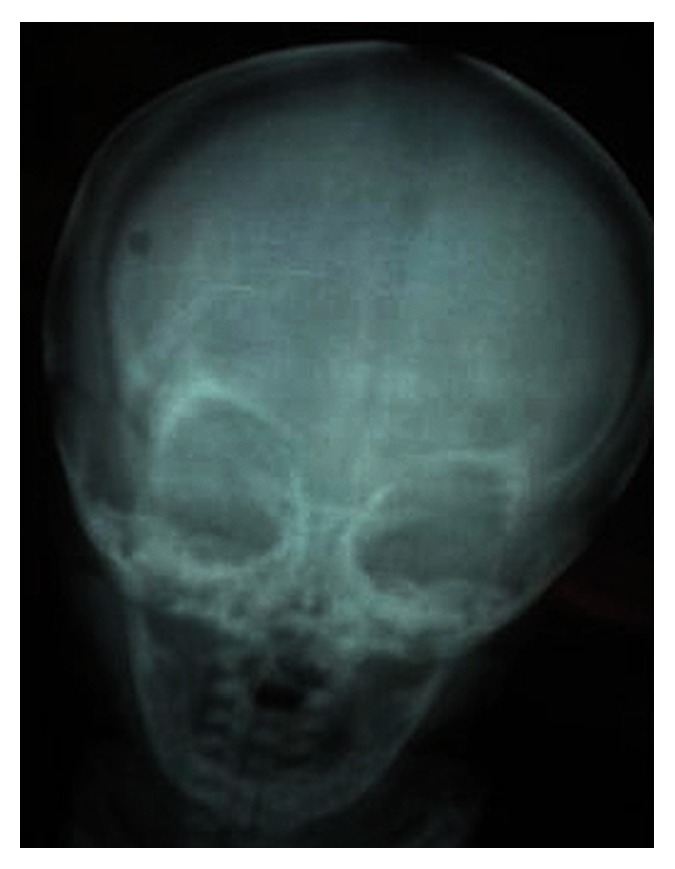
The frontal radiogram of the calvaria: increased periorbital bone density and the typical “space alien” face.

Histological examination of bone marrow biopsy specimens revealed hypocellularity, enlarged and thickened bone trabeculae with encroachment on marrow spaces, and a mosaic pattern with cartilaginous islands (Figure 1(a)). CD68 and tartrate-resistant acid phosphatase staining confirmed the presence of increased number of osteoclasts in the peritrabecular region (Figure 1(b)). In addition, the lack of expressions of MPO and glycophorin substantiated osteoclastic nature of these cells. With these histopathological features, she was diagnosed as osteopetrosis. After histopathological examination of biopsy specimens, whole body skeletal survey was performed which revealed uniformly dense, homogeneous, and sclerotic bones with absence of corticomedullary junctions. Frontal radiograms revealed enlargement of the calvaria, upward slanting of the greater sphenoid alae, increased periorbital bone density, and typical “space alien” face (Figure 2). According to physical, pathological, laboratory, and radiological findings diagnosis of MIOP was made on postnatal 26th day.

Meanwhile she continued to experience hypoglycemic episodes. Her thyroid function tests were compatible with central (hypopituitary) hypothyroidism. Baseline plasma cortisol and adrenocorticotropic hormone (ACTH) levels were lower than normal reference values. Therefore low-dose ACTH stimulation test was performed and the results were interpreted in favour of secondary adrenal insufficiency (Table 2). Pituitary radiograms and MRI raised concerns about a presumptive diagnosis of sclerotic dysplasia with intensively increased bone density of the sella turcica. Hypopituitarism secondary to osteopetrosis was diagnosed and hydrocortisone treatment was started. After corticosteroid treatment hyperbilirubinemia was resolved and effective glycemic control was obtained. Anemia and thrombocytopenia were ameliorated with packed red blood cell and platelet transfusions. She was referred for hematologic stem cell transplantation (HSCT), but she passed away because of an intrusive fungal sepsis on postnatal 52nd day.

## 3. Discussion

Osteopetrosis is a progressive bone disease characterized by abnormally functioning osteoclasts and impaired bone resorption [1–3]. It can manifest through a spectrum of symptoms of varying severity, from MIOP to benign conditions with the incidental radiographic findings in adulthood (benign autosomal dominant osteopetrosis). Disturbances of osteoclastic function due to mutations in a gene encoding an osteoclast-specific subunit of the vacuolar proton pump (TCIRG1 and OSTM1) have been detected in most patients with the recessive form. Mutations of the gene encoding the chloride channel protein (CLCN7) are observed in the dominant form [1–3, 6]. Both types of mutations lead to disturbances of acidification needed for normal osteoclastic function [1]. The majority of MIOPs are diagnosed soon after birth or within the first years of life with manifestations of severe bone marrow failure, hepatosplenomegaly, characteristic macrocephaly, bone fractures, and failure to thrive [1–3]. Abnormal bone formation and fibrous tissue replace the bone marrow space and finally hematopoietic activity decreases [2]. Extramedullary hematopoiesis occurs resulting in hepatosplenomegaly. Hemolysis resulting from hypersplenism worsens the anemia and causes prolonged unconjugated hyperbilirubinemia [1, 2]. The longitudinal growth of bones is impaired, resulting in short stature of varying degrees. Encroachment of hyperostotic bone on which causes gradual occlusion of the skull foramina which predisposes patients to cranial nerve entrapment and ultimate visual and auditory impairment, facial palsy, and difficulties with feeding and swallowing. Optic atrophy is reported in a significant number of cases in the literature [1, 6]. Combination of an increased thickness of the calvarium and decrease in cranial capacity may raise intracranial pressure and induce hydrocephalus requiring surgical intervention [1, 2, 5]. Calvarial deformities can also cause choanal atresia or stenosis that may result in severe respiratory distress requiring artificial ventilation soon after birth. In our case, sclerotic bone changes, especially calvarial abnormalities, hydrocephalus, and audiovisual impairment were remarkable. Our patient developed hepatosplenomegaly and pancytopenia at her postnatal 3rd week. Her peripheral blood smear was remarkable for hemolysis and leukoerythroblastosis. Her reticulocyte counts, serum unconjugated bilirubin, and lactate dehydrogenase levels also supported hemolysis.

Radiologic findings of osteopetrosis include diffuse sclerosis of the bones with a typical “bone in bone” appearance, “Batman sign,” metaphyseal changes, and fractures [1, 3, 7]. Whole body skeletal survey of our patient was specific for typical radiologic findings of osteopetrosis. She had uniformly dense, homogeneous, and sclerotic bones. The typical “space alien” face was remarkable on anteroposterior radiograms of the skull. Some infants with MIOP are at a risk of developing hypocalcemia with attendant tetanic seizures and secondary hyperparathyroidism [1, 2, 4]. There is also sufficient data in the current literature about infantile osteopetrosis complicated by paradoxal rickets [8]. Our patient developed tonic seizures on the second day of her life. However her concurrent laboratory tests did not support the presence of any electrolyte imbalance and her epileptiform seizures were attributed to her existing hydrocephalus.

So far, in only a few articles, the relationship of MIOP with endocrinopathies other than the disorders of bone metabolism has been reported. Cases of congenital hypothyroidism and osteopetrosis complicated with slipped capital femoral epiphysis have been already cited in the medical literature [1, 3, 4]. Since mutations in TCIRG1 gene are present in most patients with the autosomal recessively inherited MIOP, one can expect impaired glucose metabolism in such patients. However an association between osteopetrosis and hypo- or hyperglycemia has not been described yet. Unfortunately we could not perform genetic analysis in our patient. However with regard to the presence of prolonged jaundice, persistent hypoglycemia, central hypothyroidism, and ACTH stimulation test results supporting secondary adrenal insufficiency and sclerotic dysplasia of the sella turcica detected on pituitary MRI, our patient was diagnosed with hypopituitarism. As is known, congenital hypopituitarism due to specific genetic disorders accounts for nearly 20% of multiple pituitary hormone deficiencies. However the majority of cases consist of acquired hypopituitarism due to different causes. In our case, hypopituitarism presenting with central hypothyroidism and secondary adrenal insufficiency was thought to be associated with the sclerotic dysplasia of the sella turcica and anterior pituitary injury.

Until recently, the prognosis for this disorder had been uniformly dismal with death usually occurring within a few months of diagnosis. Hemopoietic stem cell transplantation (HSCT) is the only curative therapy for patients with MIOP [9, 10]. The success rate of HSCT varies depending on the type of the donor. Patients receiving grafts from HLA-identical siblings have a five-year disease-free survival rate of 73–79%, while transplantations with unrelated or mismatched donors have disease-free survival rates ranging between 13 and 45 percent [10]. Irreversible neurologic impairment occurs in early infancy despite successful HSCT [5]. Therefore HSCT is recommended as soon as the diagnosis is made, if possible before the age of 3 months. Novel treatments including in utero stem cell treatment, receptor activator of nuclear factor kappa-B ligand (RANKL) replacement therapy, and denosumab therapy for hypercalcaemia are under discussion in the current literature [9, 10]. In this respect, our patient was referred to another hospital for HSCT at postnatal 50 days but unfortunately she died because of a fungal sepsis before the application of the procedure.

In conclusion, MIOP is a progressive disease which can present through a variety of clinical manifestations. The frequency of certain clinical and laboratory features may vary depending on the underlying genetic defect. The concomitant occurrence of MIOP with endocrine disorders apart from derangements in bone metabolism is quite rare. Based on our literature review, our patient is the first newborn infant reported so far who presented with manifestations of hypopituitarism due to sclerotic dysplasia of the sella turcica and was definitively diagnosed by histopathological examination of the bone marrow biopsy specimens. In this respect we would like to emphasize the importance of careful evaluation of MIOP patients for several organ dysfunctions including central nervous system pathologies and secondary hormonal disturbances. We would also like to underline the importance of interpreting plain chest X-ray of an infant with regard to bone density since a careful examination may provide an opportunity for a rapid diagnostic and therapeutic workup. Finally since we could not perform a genetic analysis in our patient, we are not able to discuss the existence of a possible specific mutation giving rise to the unusual combination of MIOP with hypopituitarism. However we believe that further genetic studies will provide more detailed data about this interesting topic.

## Figures and Tables

**Table 1:** Laboratory features with respect to postnatal age.

Laboratory parameters	At birth	Postnatal day 17	Postnatal day 26	Postnatal day 35
Hemoglobin (g/dL)	15.4	8	7.2	7.8
Platelets (×10^9^/L)	348	74	65	52
Leukocytes (×10^9^/L)	19	8.4	5.2	4.1
Reticulocytes (%)	1.1	5.6	5.1	3.8
Serum glucose (mg/dL)	76	35	42	72
Serum total calcium (mg/dL)	8.7	8.1	7.8	7.5
Serum phosphorus (mg/dL)	6.3	5.5	5.8	6.2
Serum magnesium (mg/dL)	2.1	1.7	1.9	2.0
Serum LDH (U/L)	85	610	578	356
Serum ALP (IU/L)	160	330	570	610
Serum AST (IU/L)	32	61	102	110
Serum ALT (IU/L)	28	34	75	82
Serum total bilirubin (mg/dL)	1.16	16	15.5	10.2
Serum conjugated bilirubin (mg/dL)	0.5	0.8	4.8	2.7
Serum thyrotropin (mU/L)	—	—	0.6	0.7
Serum total thyroxine (*μ*g/dL)	—	—	7	8.2
Serum free thyroxine (ng/dL)	—	—	1.5	2.1

LDH: lactate dehydrogenase, ALP: alkaline phosphatase, AST: aspartate aminotransferase, and ALT: alanine aminotransferase.

**Table 2:** ACTH stimulation test results.

Plasma hormone levels	Baseline	60 minutes after intravenous administration of 1 *μ*g/1.73 m^2^ cosyntropin
ACTH (pg/mL)	8.3	—
Cortisol (*μ*g/dL)	1.1	18

ACTH: adrenocorticotropic hormone.
